# Förster Resonance Energy Transfer and Enhanced Emission in Cs_4_PbBr_6_ Nanocrystals Encapsulated in Silicon Nano-Sheets for Perovskite Light Emitting Diode Applications

**DOI:** 10.3390/nano14191596

**Published:** 2024-10-03

**Authors:** Araceli Herrera Mondragon, Roberto Gonzalez Rodriguez, Noah Hurley, Sinto Varghese, Yan Jiang, Brian Squires, Maoding Cheng, Brooke Davis, Qinglong Jiang, Mansour Mortazavi, Anupama B. Kaul, Jeffery L. Coffer, Jingbiao Cui, Yuankun Lin

**Affiliations:** 1 Department of Physics, University of North Texas, Denton, TX 76203, USA; araceliherreramondragon@my.unt.edu (A.H.M.); yan.jiang@unt.edu (Y.J.); jingbiao.cui@unt.edu (J.C.); 2 Department of Chemistry and Physics, University of Arkansas, Pine Bluff, AR 71601, USAjiangq@uapb.edu (Q.J.);; 3 Department of Materials Science and Engineering, University of North Texas, Denton, TX 76203, USA; 4 Department of Electrical Engineering, University of North Texas, Denton, TX 76203, USA; 5 Department of Chemistry and Biochemistry, Texas Christian University, TCU Box 298860, Fort Worth, TX 76129, USA

**Keywords:** halide perovskite, photoluminescence, Cs_4_PbBr_6_, Förster resonance energy transfer (FRET), fluorescence lifetime image (FLIM), lifetime, enhanced emission, UV exposure, nano-silicon sheets

## Abstract

Encapsulating Cs_4_PbBr_6_ quantum dots in silicon nano-sheets not only stabilizes the halide perovskite, but also takes advantage of the nano-sheet for a compatible integration with the traditional silicon semiconductor. Here, we report the preparation of un-passivated Cs_4_PbBr_6_ ellipsoidal nanocrystals and pseudo-spherical quantum dots in silicon nano-sheets and their enhanced photoluminescence (PL). For a sample with low concentrations of quantum dots in silicon nano-sheets, the emission from Cs_4_PbBr_6_ pseudo-spherical quantum dots is quenched and is dominated with Pb^2+^ ion/silicene emission, which is very stable during the whole measurement period. For a high concentration of Cs_4_PbBr_6_ ellipsoidal nanocrystals in silicon nano-sheets, we have observed Förster resonance energy transfer with up to 87% efficiency through the oscillation of two PL peaks when UV excitation switches between on and off, using recorded video and PL lifetime measurements. In an area of a non-uniform sample containing both ellipsoidal nanocrystals and pseudo-spherical quantum dots, where Pb^2+^ ion/silicene emissions, broadband emissions from quantum dots, and bandgap edge emissions (515 nm) appear, the 515 nm peak intensity increases five times over 30 min of UV excitation, probably due to a photon recycling effect. This irradiated sample has been stable for one year of ambient storage. Cs_4_PbBr_6_ quantum dots encapsulated in silicon nano-sheets can lead to applications of halide perovskite light emitting diodes (PeLEDs) and integration with traditional semiconductor materials.

## 1. Introduction

Solution-processing halide perovskites such as APbX_3_ (A = Cs, MA, FA, and PEA (FA, formamidinium; MA, methylammonium; PEA, phenylethylammonium); X = Cl, Br, and I) are very attractive due to their low-cost production and potential commercial application in pure color LED and photovoltaic devices [1,2,3,4]. A solar-cell efficiency of up to 26.1% has been reported since their discovery [5]. The thermal stability and photostability of halide perovskites pose challenges for their commercial application. Therefore, the surface passivation and encapsulation of halide perovskites in nanotubes or polymers is necessary in order to improve their stability [2,6,7,8,9,10,11,12,13,14,15]. Sargent and Grätzel’s groups have recently applied fluorinated aniliniums for an interfacial passivation for triple cation Cs_0.05_MA_0.05_FA_0.9_Pb (I_0.95_Br_0.05_)_3_ perovskite films [16]. The accelerating aging tests of halide perovskites under high temperatures and high humidity have reported a power-conversion efficiency of 24.09% and a 1560 h T85 at the maximum power point under 1 sun illumination, operating at 85 °C and 50% relative humidity [16]. For perovskite light emitting diode (PeLED) applications, the quantum efficiency of bicomponent perovskite nanocomposites can reach near 100% [17] based on the Förster resonance energy transfer (FRET) from the core CsPbBr_3_ to the CsPbI_3_ shell. FRET [18,19] has further been reported between CsPbBr_3_ and CsPbCl_3_ [20], between (PEA)_2_PbI_4_ and MAPbBr_3_ [21], and between 2D perovskites [22].

Cs_4_PbBr_6_ is a zero-dimensional perovskite that can be easily prepared as quantum dots. Quantum dots of Cs_4_PbBr_6_ have been stabilized in glass, where the photoluminescence (PL) of perovskites shifts from 519 to 503 nm [23]. The superior thermal stability [24] and photostability [25] have been reported in CsPbBr_3_-in-Cs_4_PbBr_6_ quantum dots. Highly emissive blue (470 nm) quantum dots have resulted in a high photoluminescence quantum yield of >90% in CsPbBr_3_ + Cs_4_PbBr_6_ material systems [24]. Silicon nano-sheets are 2D layers of silicon with a tunable band gap. CsPbBr_3_ isolated in silicene has been realized for PeLED applications [26]. On the other hand, Cs_4_PbBr_6_ can have a very high photoluminescence quantum yield (PLQY), which can be two orders of magnitude higher than its 3D counterpart [27]. Furthermore, it is also desired to stabilize Cs_4_PbBr_6_ in silicene and use the silicene for integration with traditional semiconductors. FRET can also be incorporated to further improve the PLQY for PeLED applications [17]. 

In this paper, nanomaterial systems of Cs_4_PbBr_6_ ellipsoidal nanocrystals and pseudo-spherical quantum dots in silicon nano-sheets have been prepared, and their PL emissions have been studied. For Cs_4_PbBr_6_ quantum dots in silicon nano-sheets, broadband emissions have been observed due to the surface trap states of Cs_4_PbBr_6_. They are replaced by stable Pb^2+^ ion emissions. For the Cs_4_PbBr_6_ nanocrystals in silicon nano-sheets, we have observed FRET of up to 87% efficiency. A three-month-old sample of Cs_4_PbBr_6_ nanocrystals in silicon nano-sheets has shown an enhanced emission five times higher during 30 min of UV laser irradiation.

## 2. Materials and Methods

Preparation of Cs_4_PbBr_6_ nanocrystals: 71.2 mg CsBr and 24.8 mg PbBr_2_ was dissolved in 0.6 mL DMSO at 80 °C in a vial. The mixture was cooled down slowly while putting it in the methanol chamber. Green Cs_4_PbBr_6_ crystals were obtained at the bottom of the vial.

Preparation of Cs_4_PbBr_6_ nanocrystals and quantum dots in silicon nano-sheets: 45 mg of Cs_4_PbBr_6_ crystals was dissolved in 0.1 mL of DMSO and mixed with a given amount of silicon nano-sheets. In low concentrations of Cs_4_PbBr_6_ in silicon nano-sheets, the prepared Cs_4_PbBr_6_ solution was diluted by 10 times. The silicon nano-sheets were prepared following the method described in the reference [28]. In brief, the silicon nano-sheets were obtained by removing calcium ions from CaSi_2_ in concentrated HCl. As examined by TEM, a different concentration of Cs_4_PbBr_6_ in silicon nano-sheets results in different crystal sizes and shapes. Cs_4_PbBr_6_ crystals in silicon nano-sheets smaller than 10 nm form a pseudo-spherical shape. We call them Cs_4_PbBr_6_ quantum dots hereafter. Cs_4_PbBr_6_ crystals in silicon nano-sheets larger than 10 nm form an ellipsoid shape with an elongation in the silicon nano-sheet plane. We call them Cs_4_PbBr_6_ nanocrystals hereafter. 

The material characterizations and crystal structures of Cs_4_PbBr_6_ crystals, silicon nano-sheets, and Cs_4_PbBr_6_ nanocrystals in silicon nano-sheets were analyzed using Rigaku Miniflex 600 X-ray diffraction (XRD) with CuKα radiation (λ = 0.15405 nm).

Structural characterization was carried out with transmission electron microscopy (TEM) and energy-dispersive X-ray (EDX) in JEOL JEM-2100 at 200 kV. A carbon-coated 200-mesh copper grid was used for the preparation of TEM samples. The solution of Cs_4_PbBr_6_ nanocrystals in silicon nano-sheets was drop-casted on the copper grid and dried in a vacuum.

The PL of the Cs_4_PbBr_6_ crystals and Cs_4_PbBr_6_ nanocrystals in silicon nano-sheets was excited using a 375 nm UV laser (CrystaLaser, Reno, NV, USA). The PL of the Cs_4_PbBr_6_ quantum dots in silicon nano-sheets and pure silicon nano-sheets was excited using a He-Cd 325 nm laser (Kimmon, Tokyo, Japan). The PL spectra were analyzed using a BaySpec SuperGamut fiber-coupled UV-NIR spectrometer.

Fluorescence lifetime imaging (FLIM), PL decay lines, and lifetime histograms were obtained using a MicroTime 200 time-resolved confocal fluorescence microscope. The measurement was recorded using a PicoQuant PicoHarp 300 time-correlated single-photon-counter. Data acquisition was performed using SymPho Time 64 software. A Picoquant 405 nm picosecond laser was used with a repetition rate of 40 MHz. A cut-off filter of 435 nm was used before the detectors. Neutral density filters were used to reduce photon counts below 10^6^. A 20× objective lens (numerical aperture NA = 0.4) was used in the confocal microscope. For the FRET measurements, filters of 490 ± 5 nm (i.e., 485–495 nm) and 530 ± 20 nm (i.e., 510–550 nm) were placed in front of the detectors. The intensity FRET was calculated using software in SymPho Time 64. 

## 3. Results

### 3.1. Material and Structural Characteristics 

**Figure 1:**
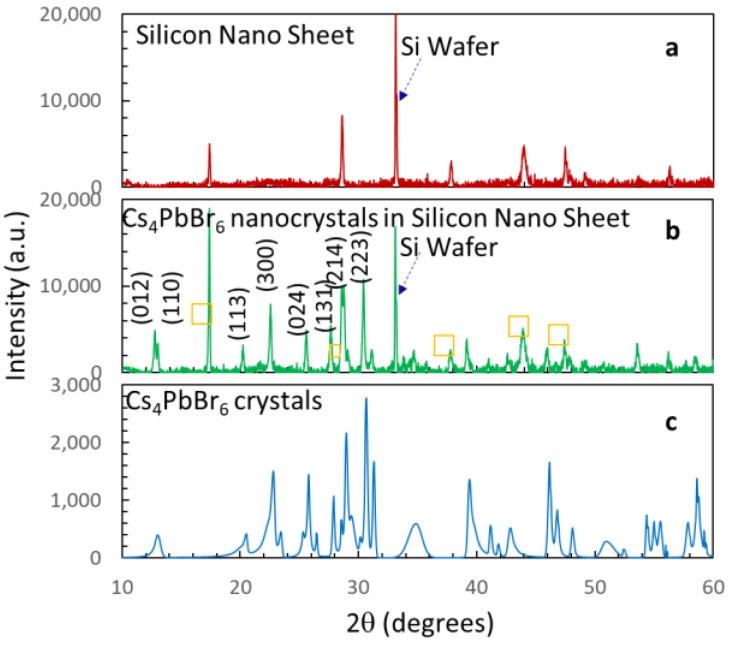
XRD patterns of silicon nano-sheets on Si wafer (**a**), Cs_4_PbBr_6_ nanocrystals encapsulated in silicon nano-sheets (**b**), and bulk Cs_4_PbBr_6_ (**c**). XRD peaks from Si wafer substrate are labeled with dashed arrows. XRD peaks from silicon nano-sheets are labeled with squares in (**b**).

The prepared solutions of silicon nano-sheets and Cs_4_PbBr_6_ nanocrystals encapsulated in silicon nano-sheets were drop-casted onto a Si wafer and dried. Figure 1 shows the X-ray diffraction (XRD) of the silicon nano-sheet, the Cs_4_PbBr_6_ nanocrystals encapsulated in silicon nano-sheets, and the bulk Cs_4_PbBr_6_ crystals. The XRD peaks at 33.1° in Figure 1a,b belong to the Si wafer, as confirmed in the supporting information in reference [26]. Silicon nano-sheets have XRD peaks at 17.4°, 28.6°, 37.8°, 44.0°, 47.5°, 49.2°, and 56.5° (Figure 1a) [26]. These peaks appear in the XRD of Cs_4_PbBr_6_ nanocrystals (high concentration) encapsulated in silicon nano-sheets in Figure 1b, as labeled with yellow squares. The XRD peaks from Cs_4_PbBr_6_ nanocrystals in Figure 1b are located at 12.6°, 12.9°, 20.1°, 22.4°, 25.4°, 27.5°, 28.6°, and 30.3°, and are assigned to the low diffraction orders of the (012), (110), (113), (300), (024), (131), (214), and (223) planes, respectively. Figure 1c shows the XRD pattern of bulk Cs_4_PbBr_6_ for comparison. The XRD pattern in Figure 1c can be fitted to the hexagonal phase of Cs_4_PbBr_6_ with a = 13.73 Å, b = 13.73 Å, c = 13.73 Å, α = 90.0°, β = 90.0°, and γ = 120.0° (XRD database Card No.: 1538416). The XRD pattern of Cs_4_PbBr_6_ quantum dots encapsulated in silicon nano-sheets shows peaks from silicon nano-sheets only due to the low concentration. However, their presence is confirmed by TEM (vide infra).

**Figure 2:**
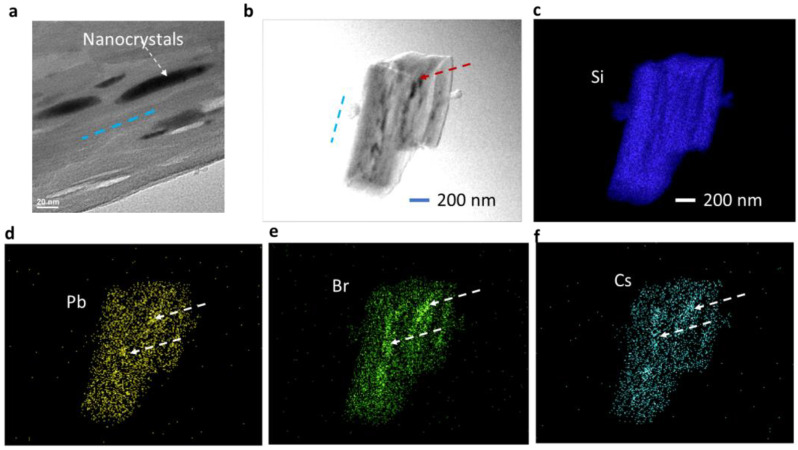
(**a**) Zoomed-in TEM image of Cs_4_PbBr_6_ nanocrystals encapsulated in silicon nano-sheets; (**b**) TEM image of one piece of sample of Cs_4_PbBr_6_ nanocrystals encapsulated in silicon nano-sheets for TEM-EDX. TEM-EDX map of Si (**c**), Pb (**d**), Br (**e**), and Cs (**f**). Cs_4_PbBr_6_ nanocrystals are pointed out by dashed arrows in (**a**,**b**). Same scale bar is used for (**b**–**f**). White arrows in (**d**–**f**) are for region with strong intensity. Dashed lines in (**a**,**b**) indicate nano-sheet orientation.

Figure 2a shows a zoomed-in image (enlarged view) of a TEM image of Cs_4_PbBr_6_ ellipsoidal nanocrystals encapsulated in silicon nano-sheets. The dashed arrow in the figure indicates the location of one Cs_4_PbBr_6_ nanocrystal (dark region). All Cs_4_PbBr_6_ nanocrystals are elongated with the same orientation, as indicated by a dashed blue line, assuming they are in the same plane as a given silicon nano-sheet. From Figure 2a, we can observe that the nanocrystal size is not uniform; the elongated nanocrystals have a larger size in the middle than these at both sides of elongation. The stretching of the nanocrystals and different sizes along the long nanocrystals will be modeled for the explanation of the multiple peaks in the PL measurements.

Figure 2b shows a TEM image of one piece of sample for TEM-EDX study. The scale bars in2(b–f are the same. The dashed blue line indicates the nano-sheet orientation. One of the dark regions is indicated by an arrow. These nanocrystals in the dark regions are in the same orientation. Over 10 of them are revealed on the surface using TEM. Inside the bulk sample, TEM-EDX maps can reveal more information. The distribution of elements associated with the Cs_4_PbBr_6_ nanocrystals encapsulated in silicon nano-sheet perovskites (i.e., Si, Pb, Br, and Cs) is shown in Figure 2c–f, respectively. The elements of Si, Pb, Br, and Cs cover the whole piece of the sample in Figure 2b, as judged by the map shape. It is understandable that the map intensity of Si in Figure 2c is uniform due to the role of the host. The map intensity of Pb, Br, and Cs is not uniform. Especially in Figure 2e, it is very clear to see that the strong intensity regions (indicated by dashed white arrows, for example) correspond to the dark regions in Figure 2b. 

**Figure 3:**
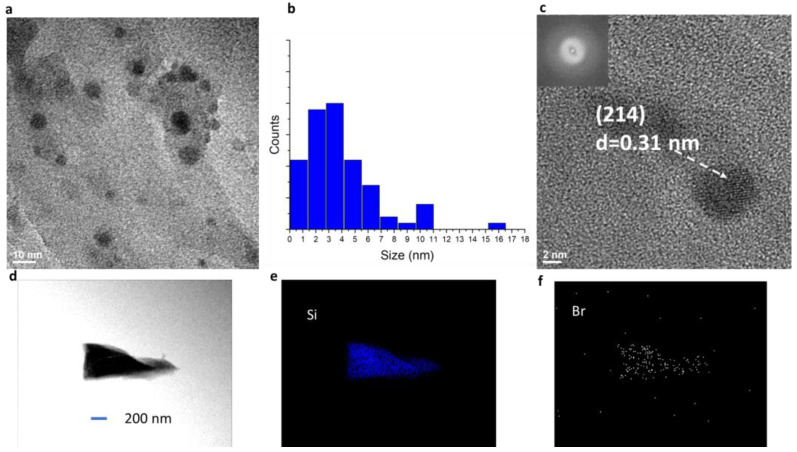
(**a**) TEM image of Cs_4_PbBr_6_ quantum dots encapsulated in silicon nano-sheets; (**b**) size distribution of Cs_4_PbBr_6_ quantum dots encapsulated in silicon nano-sheets. (**c**) High resolution TEM (HRTEM) image of Cs_4_PbBr_6_ quantum dots and corresponding FFT. TEM of one piece of sample (**d**) and its TEM-EDX map of Si (**e**) and Br (**f**). Same scale bar is used for (**d**–**f**).

The diffraction peaks from Cs_4_PbBr_6_ quantum dots did not show up in the XRD pattern due to their low concentration (diluted by 10 times). TEM allowed us to observe the quantum dots. Figure 3a shows a typical TEM image of Cs_4_PbBr_6_ pseudo-spherical quantum dots encapsulated in silicon nano-sheets. The size of the quantum dots is not uniform. Figure 3b shows the size distribution of the quantum dots in a range of 1 to 16 nm. The mean size is 4.67 nm. Figure 3c shows a high-resolution TEM (HRTEM) image of quantum dots and fast Fourier transform (FFT) patterns. A d-value of 0.31 nm was obtained, corresponding to the space between the (214) plane of hexagonal Cs_4_PbBr_6_. Figure 3d shows a TEM image of one piece of the sample and its distribution of elements associated with the Cs_4_PbBr_6_ quantum dots encapsulated in silicon nano-sheet perovskites in Figure 3e,f for Si and Br as an example, respectively. Si covers the whole sample in Figure 3e, while the map intensity of Br is not as dense as Si due to the low concentration of quantum dots.

### 3.2. PL Spectra and Förster Resonance Energy Transfer

**Figure 4:**
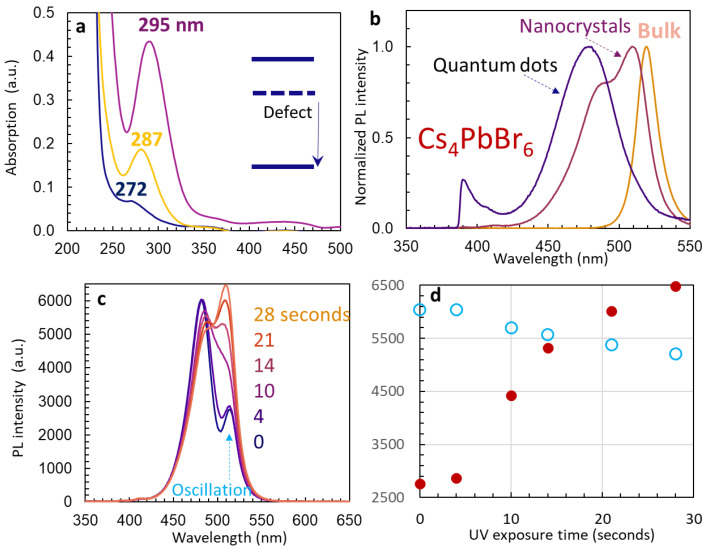
(**a**) Absorption of bulk Cs_4_PbBr_6_ (pink line), Cs_4_PbBr_6_ nanocrystals (yellow line), and quantum dots (blue line) in DMSO solution. (**b**) Normalized PL spectra of bulk Cs_4_PbBr_6_ (yellow line), Cs_4_PbBr_6_ nanocrystals (pink line), and quantum dots (blue line) encapsulated in silicon nano-sheets. (**c**) PL spectra of Cs_4_PbBr_6_ nanocrystals encapsulated in silicon nano-sheets at 0, 4, 10, 14, 21, and 28 s after UV excitation laser is turned on. (**d**) PL intensity at two wavelengths (512 nm in red and 490 nm in blue) as a function of UV exposure time.

Firstly, we measured the absorption of bulk Cs_4_PbBr_6_ Cs_4_PbBr_6_ nanocrystals and quantum dots in DMSO solution before they were mixed with silicon nano-sheets, where narrow peaks were observed at 295, 287, and 272 nm, respectively. We then measured the PL for bulk Cs_4_PbBr_6_, its ellipsoidal nanocrystals, and pseudo-spherical quantum dots encapsulated in silicon nano-sheets. Figure 4b shows normalized PLs, with a peak position at 520 nm for bulk Cs_4_PbBr_6_, two peaks at 512 and 490 nm for Cs_4_PbBr_6_ nanocrystals, and a broad peak centered at 480 nm for Cs_4_PbBr_6_ quantum dots. With a small crystal size, the PL peak is blueshifted, similar to what has been observed in CsPbBr_3_ in silicene and bulk CsPbBr_3_. The broad peak of Cs_4_PbBr_6_ quantum dots in Figure 4b can be due to the un-passivated surfaces and its surface state. Similar to other results [29], Cs_4_PbBr_6_ had a very small spectral overlap of absorption and PL emission with a Stokes shift of up to 1.82 eV. Defect states due to Br-poor vacancy or local deformation of [PbBr_6_]^4−^ octahedra were used to explain the large Stokes shift [29,30,31,32]. Although the shift of the narrow peak with the crystal size can be observed in Figure 4a, such a large Stokes shift poses a complexity in explaining the FRET below in Cs_4_PbBr_6_ in silicon nano-sheets. 

The PL in Figure 4b for Cs_4_PbBr_6_ nanocrystals in silicon nano-sheets was measured after several minutes of UV exposure. In the short period of UV 375 nm laser exposure, the peak at 512 nm is very weak initially, then increases its intensity from 0 to 28 s, and finally its intensity is higher than the peak at 490 nm, as shown in Figure 4c. We also recorded a Video S1, “PL peak intensity oscillation”. We can see the increase in peak intensity at 512 nm, turning off the UV laser at 36–42 s, the dropping of the peak intensity, and its increase again after the UV laser is turned on. Figure 4d shows the peak intensity as a function of UV exposure time, where we see an increase in intensity by more than two times for 512 nm emission in 20 s. 

**Figure 5:**
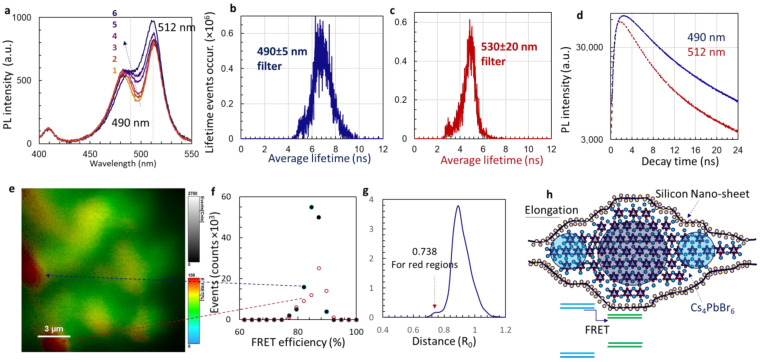
(**a**) PL spectra measured after Cs_4_PbBr_6_ nanocrystals encapsulated in silicon nano-sheets were exposed to UV 375 nm laser for several minutes. Six of them were measured starting with “1” and ending with “6”. (**b**,**c**) Average PL lifetime histogram measured with bandpass filters of 490 ± 5 nm (i.e., 485–495 nm) (**b**) and 530 ± 20 nm (**c**). (**d**) PL intensity decay curves for 490 and 512 nm. (**e**) Intensity and efficiency of FRET and (**f**) FRET efficiency histogram for two red regions in (**d**) as indicated by dashed arrows. (**g**) Occurred FRET events at different distances. (**h**) Schematic of elongated Cs_4_PbBr_6_ nanocrystals encapsulated in silicon nano-sheets. Circles show different crystal sizes. Different sizes lead to different bandgaps for FRET.

In order to understand both the intensity oscillation of the 512 nm emission peak and the exchange of peak intensity of both PL lines in Cs_4_PbBr_6_ nanocrystals encapsulated in silicon nano-sheets, we measured the lifetime of PL peaks and FRET in the next steps. Due to the intensity change and shift of the PL peaks with UV laser exposure time, we measured the PL after the UV laser had been turned on for several minutes (typical time for PL lifetime imaging). We measured the PL for six times of the irradiation hardening, starting from yellow to dark blue as shown in Figure 5a. The irradiation-hardened Cs_4_PbBr_6_ nanocrystals had PL peaks at 490 and 512 nm. We used bandpass filters of 490 ± 5 nm (i.e., 485–495 nm) and 530 ± 20 nm (i.e., 510–550 nm) and measured the FLIM. After fitting and calculation, Figure 5b,c show the average lifetime histogram for the PL peaks at 490 and 512 nm, respectively. The lifetime events were controlled below 10^6^ for measurement accuracy. As seen from Figure 5b,c, the average lifetime is centered at 6.8 ns for 490 nm and 5.0 ns for 512 nm. Their PL decay lines are shown in Figure 5d. As the nanocrystal size becomes smaller, the PL peak usually blueshifts [33]. The PL of 490 nm comes from a smaller nanocrystal than that of 512 nm. The more surface localized charges [34] (also the more surface trap states) in smaller nanocrystals, the higher the PL lifetime, which also makes the lifetime histogram curve broader in Figure 5b than that in Figure 5c.

Using bandpass filters of 490 ± 5 nm and 530 ± 20 nm, we measured the FLIM and calculated FRET events and efficiency between 490 nm and 512 nm as shown in Figure 5e. The FRET efficiency E [19] is defined in Equation (1):

(1) $$E=\frac{1}{{\left(\frac{R}{R_{0}}\right)}^{6}+1}$$

where *R_o_* is the distance between the Cs_4_PbBr_6_ donor (490 nm emission) and the Cs_4_PbBr_6_ acceptor (512 nm emission) with a FRET efficiency of 50%, and *R* is the distance between the donor and the acceptor. Non-uniformity in the FRET efficiency is clearly observed. Based on the scale bar, most of areas are covered by a green color with approximately 66% efficiency with a distance of 0.89 *R_o_*_,_ as also calculated by Equation (1). For the two red regions, we processed with the “region of interest” and obtained a FRET efficiency histogram in Figure 5f. Both red regions have a FRET efficiency of 86–87%, corresponding to a distance of 0.738 *R_o_*_,_ as indicated by the red arrow in Figure 5g, where the occurred FRET events over the map region are plotted with respect to *R*. Figure 5h shows a schematic of the proposed model of Cs_4_PbBr_6_ nanocrystals encapsulated in silicon nano-sheets with horizontal elongation, following the information that was obtained using TEM in Figure 2a,b for Cs_4_PbBr_6_ ellipsoidal nanocrystals in the dark regions. The circles in Figure 5h are for visual indication only. We have a schematic of wide (blue lines) and narrow (green lines) bandgaps for the Cs_4_PbBr_6_ nanocrystals above. FRET can occur between these wide and narrow bandgaps. 

### 3.3. Stability of Un-Passivated Cs_4_PbBr_6_ and Enhanced PL Emissions

**Figure 6:**
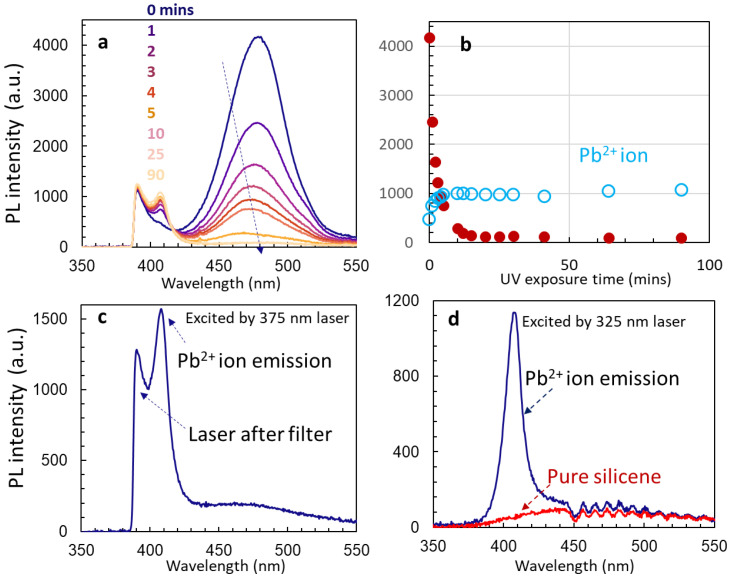
(**a**) PL spectra of Cs_4_PbBr_6_ quantum dots encapsulated in silicon nano-sheets after different lengths of UV laser exposure of 0, 1, 2, 3, 4, 5, 10, 25, and 90 min; (**b**) PL intensities as a function of UV laser exposure time for 483 nm (red circles) and Pb^2+^ ion emission at 410 nm; (**c**) PL of Pb^2+^ ion emission after UV degradation of Cs_4_PbBr_6_ quantum dots for 5 h. (**d**) PL of degraded Cs_4_PbBr_6_ quantum dots and pure silicene, excited using UV 325 nm laser.

We present the stability of un-passivated Cs_4_PbBr_6_ quantum dots encapsulated in silicon nano-sheets, their non-uniformity, their Pb^2+^ ion emission, and their enhancement of 512 nm emission. Figure 6a shows the PL spectra of Cs_4_PbBr_6_ quantum dots encapsulated in silicon nano-sheets after continuous UV laser exposure for 0, 1, 2, 3, 4, 5, 10, 25, and 90 min. The broad peak centered at 483 nm drops in intensity very quickly within 5 s. The intensity of Pb^2+^ ion emission [31,32] at 410 nm increases quickly with increasing exposure. Figure 6b shows the PL intensities as a function of UV laser exposure time for these two peaks. At 12 min, the intensity of the 483 nm peaks reaches almost zero, while the Pb^2+^ ion emission reaches the maximum at 12 min and is stable up to the end of measurement (90 min), indicating that un-passivated Cs_4_PbBr_6_ quantum dots are not stable, and isolated Pb^2+^ ions are formed and are stable. After a longer UV exposure time, Pb^2+^ ion emission becomes stronger. Figure 6c shows the PL of Pb^2+^ ion emission after the UV degradation of Cs_4_PbBr_6_ quantum dots for 5 h. The peak below 400 nm is the emission tail of the 375 nm laser after a band cutoff filter. To confirm this, we excited the PL of degraded Cs_4_PbBr_6_ quantum dots using a UV 325 nm laser. Figure 6d shows the PL of the degraded Cs_4_PbBr_6_ quantum dots with one peak only, at 410 nm. We also show the PL spectrum of pure silicene, excited using a UV 325 nm laser for comparison. The silicene (silicon nano-sheets) has no PL between 350 and 550 nm. 

**Figure 7:**
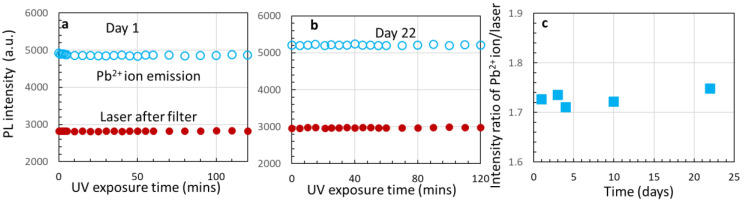
The PL intensity of Pb^2+^ ion emission and laser tail intensity after a filter on day 1 (**a**) and day 22 (**b**). (**c**) The laser intensity is stable and is used as a reference for the intensity ratio of Pb^2+^ ion emission on different days.

We further studied the stability of Pb^2+^ ion emission. We picked up degraded Cs_4_PbBr_6_ quantum dots and monitored the intensity stability of the 410 nm peak (Pb^2+^ ion emission [31,32]) as shown in Figure 7a on day 1. The red circles in the figure indicate that the laser power is very stable. The intensity of Pb^2+^ ion emission changed slightly at the very beginning and then became stable. We measured the intensity of Pb^2+^ ion emission on days 3, 4, 10, and 22 for 2 h. Figure 7b plots the intensity of Pb^2+^ ion emission on day 22 as a representative example, and Figure 7c shows the intensity ratio of Pb^2+^ ion emission over the laser tail power. Overall, the intrinsic Pb^2+^ ion emission is stable.

**Figure 8:**
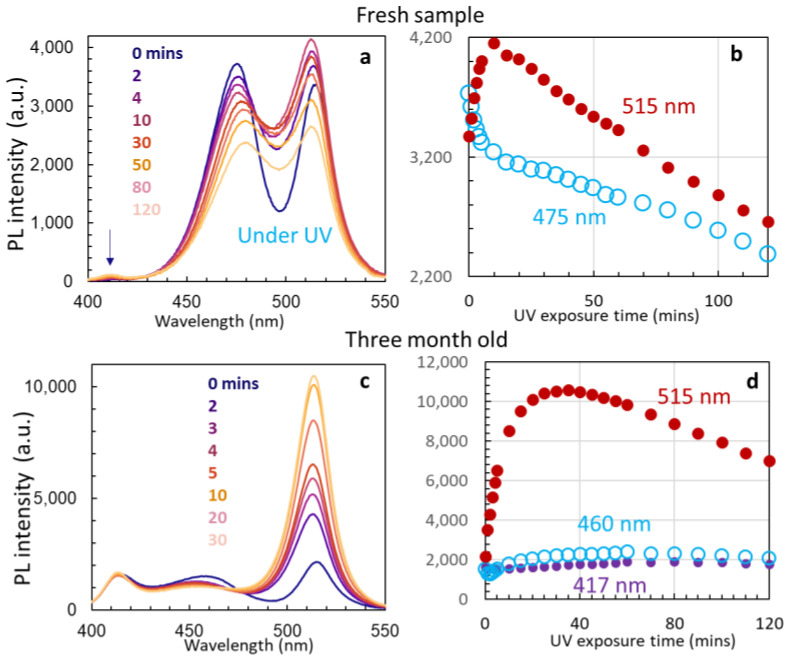
(**a**) PL spectra in a region of the sample where Cs_4_PbBr_6_ nanocrystals have three PL peaks at 515 nm, 475 nm, and the weak 410 nm line (as indicated by an arrow) after 0, 2, 4, 10, 30, 50 80, and 120 min of UV 375 nm laser exposure; (**b**) PL intensities of 515 nm and 475 nm as a function of length of UV 375 nm laser exposure. (**c**) PL spectra from 3-month-old sample after 0, 2, 3, 4, 5, 10, 20, and 30 min of UV 375 nm laser exposure; (**d**) PL intensities of 515 nm, 460 nm, and 417 nm as a function of length of UV 375 nm laser exposure.

With the above understanding of the stability of Pb^2+^ ions and the instability of Cs_4_PbBr_6_ quantum dots, we further studied a region of the sample where Cs_4_PbBr_6_ nanocrystals have three PL peaks at 515 nm, 475 nm, and 410 nm, as shown in Figure 8a. The shift of the 490 nm peak in Figure 5a to 475 nm in Figure 8a indicates smaller nanocrystal sizes in Figure 8a than that in Figure 5a. From Figure 8a,b, we can learn that the intensity of the PL peak of 515 nm is initially weaker than that of 475 nm, it increases at the very beginning of UV 375 nm exposure, it becomes stronger than that of 475 nm, and then it decreases in intensity with increasing exposure time. For the 475 nm peak, its intensity drops quickly at the very beginning of UV exposure and drops at a slower rate together with the 515 nm peak in Figure 8b. The dropping of intensity of both PL peaks can be caused by the instability of Cs_4_PbBr_6_ nanocrystals related to the 475 nm peak. 

We waited for 3 months for the disappearance of the 475 nm peak. As shown in Figure 8c, the 3-month-old sample has a weak peak at 460 nm. However, the Pb^2+^ ion emission becomes relatively strong due to the degradation of Cs_4_PbBr_6_ nanocrystals. In such an aged sample, the intensity of PL 515 nm increases five times upon exposing the sample to a UV 375 nm laser for 15 min, and reaches the maximum after 30 min, as shown in Figure 8c,d. The Pb^2+^ ion emission at 417 nm was stable, and the intensity of 460 nm varied slightly. The stable PL of Pb^2+^ ion emission can lead to the possibility of photon recycling (the process of photon re-absorption and internal re-emission from Pb^2+^ to 515 nm) for the enhancement of 515 nm. Future research can be performed on the passivation of Cs_4_PbBr_6_ nanocrystals, then further encapsulated in silicon nano-sheets for photoelectric devices.

**Figure 9:**
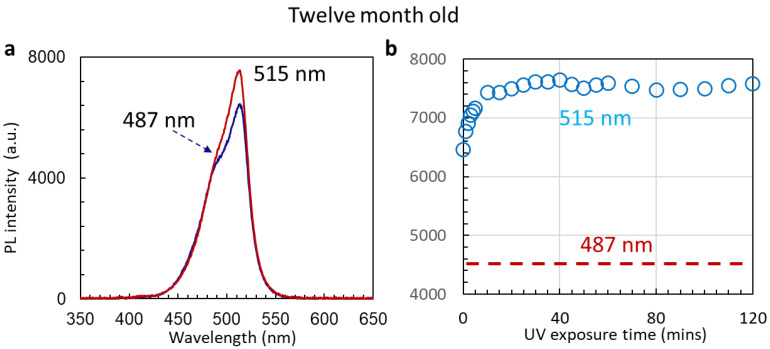
(**a**) PL spectra of 12-month-old sample of Cs_4_PbBr_6_ nanocrystals encapsulated in silicon nano-sheets at 0 (blue line) and 120 min (red line) after UV excitation laser is turned on. (**b**) PL intensity at 515 nm as function of UV exposure time. We did not conduct peak intensity fitting for 487 nm. The dashed red line is for visual indication only.

After 12 months of ambient storage of Cs_4_PbBr_6_ nanocrystals in silicon nano-sheets, we were still able to observe the FRET, as shown in Figure 9. This time, the peak intensity at 487 nm was stable during the measurement period from 0 to 120 min. The peak intensity at 515 nm was stronger than the 487 nm peak. Its intensity increased from 6400 to 7600 in a short time, then stayed almost constant. By the comparison of two peak intensities in Figure 9a,b, we can see that FRET plays a crucial role in the PL enhancement of 515 nm for PeLED application.

## 4. Discussion

**Figure 10:**
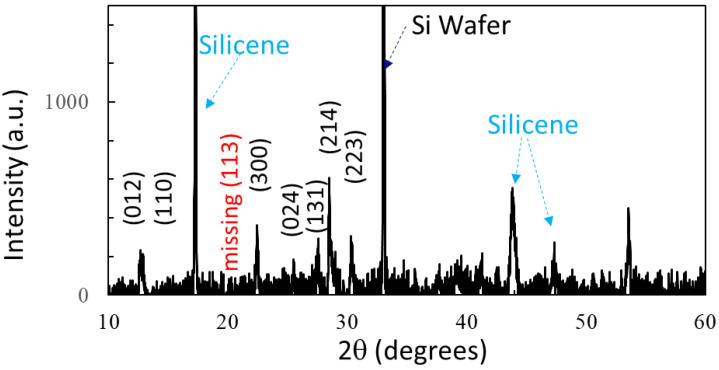
XRD patterns of Cs_4_PbBr_6_ nanocrystals encapsulated in silicon nano-sheets that are 12 months old.

Cs_4_PbBr_6_ was reported to be stable under alpha-particle excitation [35]. We first discussed the UV irradiation effect on Cs_4_PbBr_6_ nanocrystals encapsulated in silicon nano-sheets. After 12 months, we rechecked XRD for the sample as shown in Figure 10. As we can see from the figure, XRD peaks from silicon nano-sheet were still observed, indicating silicon nano-sheets were stable for 12 months. We can still observe the XRD peaks from Cs_4_PbBr_6_ nanocrystals at 12.6°, 12.9°, 22.4°, 25.4°, 27.5°, 28.6°, and 30.3° due to the low diffraction orders of the (012), (110), (300), (024), (131), (214), and (223) planes, respectively. Diffraction from the (113) plane is missing and XRD peaks are weaker from the aged sample than the fresh one, corresponding to the fact that the broad peak around 460 nm from the Cs_4_PbBr_6_ became weak and Pb^2+^ emission appeared after UV irradiation in Figure 6 and Figure 8.

**Figure 11:**
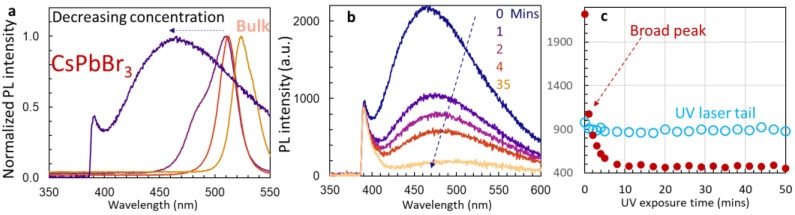
(**a**) Normalized PL spectra of CsPbBr_3_ with decreasing concentration in silicon nano-sheets. (**b**) PL spectra of CsPbBr_3_ quantum dots encapsulated in silicon nano-sheets after different lengths of UV laser exposure of 0, 1, 2, 4, and 35 min; (**c**) PL intensities as function of UV laser exposure time for broad peak centered at 466 nm.

We further discussed why the PL peak from Cs_4_PbBr_6_ quantum dots is broader than those from nanocrystal or bulk material. Usually, the PL peak becomes narrower when the nanocrystal size becomes smaller due to the quantum discrete state [33]. We assume that there are many surface trap states in the interface of silicon nano-sheets and un-passivated quantum dots, leading to a broad PL peak. To confirm this assumption, we compared our results for Cs_4_PbBr_6_ with those for CsPbBr_3_. We prepared CsPbBr_3_ encapsulated in silicon nano-sheets with decreasing concentrations (the quantity of the concentration was ignored) and measured their PL as shown in Figure 11a. Similarly, a broad peak was observed at the low concentration of CsPbBr_3_ in silicon nano-sheets. The intensity of the broad peak dropped quickly during the UV 375 nm laser irradiation, as shown in Figure 11b,c. The only difference between the Cs_4_PbBr_6_ and CsPbBr_3_ samples is that there is no Pb^2+^ ion emission in the CsPbBr_3_ sample. 

## 5. Conclusions

Silicon nano-sheets have been used to encapsulate Cs_4_PbBr_6_ nanocrystals for their potential integration with traditional semiconductors. For low concentrations of un-passivated Cs_4_PbBr_6_ in silicon nano-sheets, pseudo-spherical quantum dots were formed with a broad PL peak, which was not stable upon irradiation with a UV 375 nm laser. The Pb^2+^ ion emission was stable upon the degradation of Cs_4_PbBr_6_ quantum dots. For high concentrations of un-passivated Cs_4_PbBr_6_ in silicon nano-sheets, ellipsoidal nanocrystals were observed. The intensity oscillation of two PL peaks was observed upon the UV laser being switched on and off after exposure, and explained by FRET with an efficiency of up to 86–87%. In a sample with three PL peaks at 515, 475, and 410 nm, the degraded (aged for 3 months) sample showed stable Pb^2+^ ion/silicene emission and enhanced intensity by five times for 515 nm, presumably due to photon recycling from Pb^2+^ ion/silicene emission to 515 nm.

## Data Availability

The data will be available upon request.

## Supplementary Materials

The following supporting information can be downloaded at https://www.mdpi.com/article/10.3390/nano14191596/s1, Video S1: PL peak intensity oscillation.
